# Pancreatitis following bariatric surgery

**DOI:** 10.1186/s12893-019-0532-6

**Published:** 2019-07-05

**Authors:** Kiran Chandni Baran, Maurits de Brauw

**Affiliations:** 1 Resident Burn Medicine, Burn Center beverwijk, Red Cross hospital, Beverwijk, The Netherlands; 2 Bariatric Surgeon, Department of surgery, Spaarne Gasthuis Hoofddorp, Beverwijk, The Netherlands

**Keywords:** Acute pancreatitis, Bariatric surgery, Ileus, Small bowel obstruction, Obstructing blood clot

## Abstract

**Background:**

The laparoscopic Roux-en-Y gastric bypass (LRYGBP) is the second most performed bariatric surgical procedure. With the increasing number of patients undergoing bariatric surgery, the number of complications is also growing. Early diagnosis and treatment of the complications is crucial.

**Case presentation:**

A very unusual complication was met after an uneventful laparoscopic gastric bypass (LGBP) procedure due to an obstructing blood clot in the biliairy limb resulting in an acute pancreatitis and gastric distention, accompanied by an obstructing blood clot in the distal ileum causing small bowel obstruction. A review of the occurrence of these complications and the diagnosis and treatment is presented.

**Conclusion:**

Post-bariatric acute pancreatitis is uncommon, but could be fatal. Blood clots should be considered as possible causes of small bowel obstruction, ileus or pancreatitis.

## Key points

- Pancreatitis shortly after bariatric surgery is unusual but can be fatal.
- Post-bariatric pancreatitis may be caused by stasis due to small bowel obstruction.
- An intraluminal hematoma should be considered in the differential diagnosis of post-operative small bowel obstruction.
- Hemostasis during surgery is of significant importance.
- Early diagnosis and treatment of pancreatitis is necessary.

## Background

The number of performed bariatric surgeries is increasing [1, 2]. The laparoscopic Roux-en-Y gastric bypass (LRYGBP) is the second most performed bariatric procedure with a low mortality and morbidity [2, 3]. Frequently described complications are anastomosis related, such as bleeding, leakage, stenosis of the anastomosis or intestinal obstruction [4]. However, pancreatitis directly following bariatric surgery is very uncommon. Pancreatitis mostly resolves without complications (80%), but in the case of a severe pancreatitis (20%), the complications can be fatal and result in death (3%) [5, 6]. A rare combination of complications following blood clots is described.

## Case presentation

A 33-year-old female presented to the emergency room with progressive abdominal pain. The patient underwent a LRYGBP two days prior to her admission. The primary operation was uneventful. Our technique involves double stapling of the intestinal jejunal-jejunal anastomoses using two 60 mm 2.5 mm staplers. No bleeding problem was encountered during this operation. Postoperative, she received subcutaneous low-molecular weight heparin for one week. She did not have a relevant past history. Her current medications were citalopram, pantoprazole and nadroparin.

The abdominal pain had a sudden onset and increased gradually. The patient had continuous severe abdominal pain localized in the left hemi-abdomen, intensifying from time to time (colic). Other complaints were nausea and vomiting. Since the LGBP, the patient did not have any stool. Flatulence was present. During physical examination, the patient experienced a lot of pain. Temperature, heart rate and blood pressure were normal. The bowel sounds were high pitched during auscultation. Palpation of the abdomen was mostly tender in the left hemi-abdomen. Biochemical analysis showed a C reactive protein (CRP) of 47 mmol/L, white cell count of 19.0 × 10 9/L, a glomerular filtration rate of more than 90 and a lipase of 47 U/L.

**Fig. 1:**
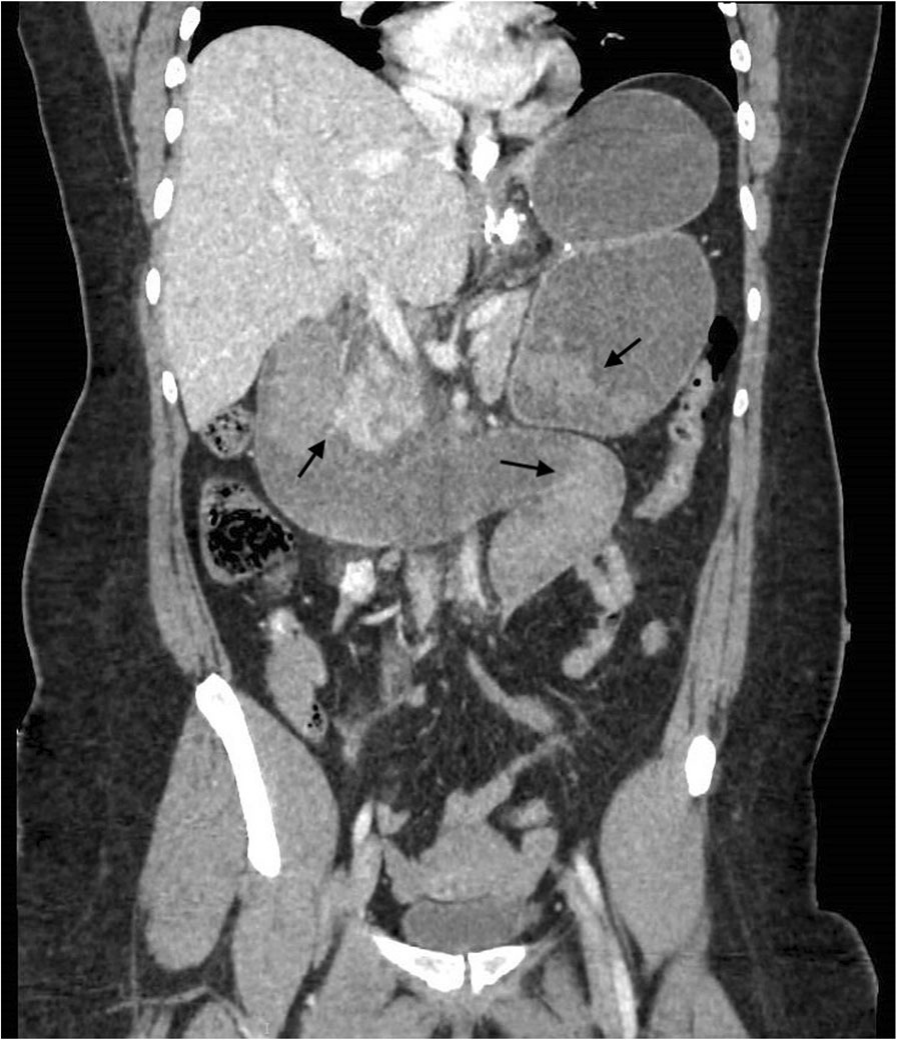
CT abdomen on the day of presentation. The CT-scan shows dilatation of the excluded stomach and of the proximal small intestines with hyperdensity (arrow), which appeared to be intraluminal blood clots during laparoscopy

An abdominal CT-scan showed severe dilatation of the excluded stomach, filled with fluid. The whole trajectory of the proximal small intestines was distended, up to the Y-anastomosis. Remarkable was that the more distal small bowel was also distended, almost up to the distal ileum. A hyperdensity was seen in the excluded stomach and in the proximal small intestines (Fig. 1).

A laparoscopy was performed. During laparoscopy, an intraluminal obstruction was found proximal of the jejunojejunostomy, causing dilatation of the excluded stomach and biliairy limb. Both, the alimentary limb as well as the common channel were distended due to a bulk found at the distal ileum. The bulk was movable trough gentle massage into the colon. The mass was a large blood clot, which appeared as a hyperdensity on the abdominal CT scan (Fig. 1, arrow). Another obstructing blood clot in the distal biliairy limb was removed by opening the blind loop of this limb and removing the cause of obstruction. The severely distended excluded stomach was decompressed with diathermic perforation and suction, followed by closing the defect with a V-lock.

Postoperatively, the patient had a persisting tachycardia, severe abdominal pain and elevated infection parameters (CRP 455 mmol/L; white cell count of 37.8 0 × 10 9/L) and a lipase of 207 U/L. Another laparoscopy was performed, which showed no signs of anastomotic leakage. A pancreatitis was seen: the pancreas was edematous and the pancreatic body was enlarged. Treatment was the standard management of pancreatitis.

The acute pancreatitis had progressed with a lipase of 697 U/L. The patient developed fever, for which intravenous ceftriaxone and metronidazole was given.-

An ultrasonography of the abdomen showed no cholelithiasis as a cause of pancreatitis.

Repeated CT-scan of the abdomen showed an edematous pancreas and peripancreatic fat infiltration, without any sign of pancreatic necrosis or intra-abdominal abcess.

The symptoms of the patient improved during the course of her admission and the inflammation parameters normalized. She was discharged after 16 days.

## Discussion and conclusions

Due to the increasing number of performed LRYGBP, clinicians may notice a rising number of its complications [1–3]. Early post-operative complications are anastomosis-leakage, gastrointestinal bleeding and small bowel obstruction [7]. Internal herniation, anastomotic strictures and marginal ulcerations occur in the long term [8].

Pancreatitis shortly after bariatric surgery is very uncommon in recent literature. One case of a fulminant pancreatitis after LRYGBP has been described, which resulted in death. The pancreatitis occurred 31 h after the procedure. A laparoscopy showed a blood clot in the jejunojejunostomy, causing obstruction of the alimentary and biliary limb [9]. A case report described acute pancreatitis after a Roux-en-Y gastric bypass due to reflux into the biliairy limb, however diagnostics did not show any sign of small bowel obstruction [10].

In a study on acute pancreatitis following bariatric surgery, the mean time-frame for developing pancreatitis was 3,5 years after bariatric surgical procedures [11].

A study reviewed retrospectively the effects of bariatric surgery on the outcome of acute pancreatitis. Gallstones have been found to be associated with post-bariatric pancreatitis [12].

Our patient did not have gallstones on ultrasonography. Three days prior to the pancreatitis, our patient underwent bariatric surgery, which was complicated by a small bowel obstruction (on CT-scan). During laparoscopy, we found an intraluminal hematoma in the jejunojejunostomy, causing intestinal stasis and dilatation of the small intestines and excluded stomach.

We hypothesize that stasis and reflux of gastrointestinal content, bile and pancreatic secretions caused the pancreatitis in our patient. Due to increasing pressure from an occluding blood clot distally in the small bowel, intestinal content retrogradely flowed into the biliairy limb, through the papilla of Vater into the pancreas. This probably activated the pancreatic enzymes, which explains elevated lipase, and resulted in a pancreatitis. Therefore, elevated serum pancreatic enzymes in bariatric patient should be given immediate attention.

Small bowel obstruction is a known complication of LGBP with an incidence of 1.9–7.3% [13, 14]. Common causes of small bowel obstruction are internal herniations, incarcerated port-site hernia, stenosis of the anastomosis, adhesions and intussusception. It is a well-known phenomenon that blood chemistry shows an elevated lipase concentration, in case of small bowel obstruction [15, 16]. Also pancreatitis has been described as sign of obstruction of the biliary limb due to internal herniation [15, 17, 18].

An intraluminal hematoma causing postoperative small bowel obstruction is a rare event [7]. We suppose that the blot clot was caused by bleeding from the staple line of the jejunojejunostomy or an intraluminal small vessel hemorrhage, since no bleeding was seen during the LGBP. Obtaining hemostasis during surgery is of significant importance.

Our patient used postoperatively prophylactic low-molecular weight heparin 2850 IU, which may have worsened the intraluminal bleeding. However, preventing venous thromboembolism in bariatric patients is of great importance and should not be passed [19].

In conclusion: pancreatitis is an uncommon short-term complication of bariatric surgical procedures. Post-bariatric pancreatitis may be caused by stasis following early small bowel obstruction. Elevated serum lipase or amylase could be a sign of stasis and thus the beginning of a pancreatitis. Most cases of pancreatitis are self-limiting, however, severe pancreatitis could be fatal. In case of intestinal obstruction and ileus and or pancreatitis, blood clots should be considered as possible causes.

## Data Availability

All data is contained within the manuscript and its additional files.

## Abbreviations

CT: Computed tomography
LGBP: Laparoscopic gastric bypass
LRYGBP: Laparoscopic Roux-en-Y gastric bypass
